# Intravitreal Faricimab for the Management of Bilateral Macular Neovascularization Secondary to Macular Telangiectasia Type 2

**DOI:** 10.7759/cureus.92696

**Published:** 2025-09-19

**Authors:** Prasan Rao, Anupama Rao, Jabeen Mohammed Jaffer, Ansu Sara John

**Affiliations:** 1 Ophthalmology, Medcare Eye Centre, Dubai, ARE

**Keywords:** ellipsoid zone integrity, faricimab, intraretinal cavitation, macular neovascularization, macular telangiectasia type 2

## Abstract

Macular telangiectasia type 2 (MacTel2) is a bilateral neurodegenerative disease characterized by telangiectasia of macular vasculature and foveal atrophy that is typically acquired during middle age. It may lead to visual loss due to progressive reduction of photoreceptor function or secondary to macular neovascularization. We report a case of bilateral macular neovascularization associated with MacTel2, which was treated with intravitreal faricimab injections. A 52-year-old female presented with a history of progressive reduction of vision in both eyes. Dilated fundus examination revealed the presence of perifoveal telangiectasia and right-angled venules in the juxtafoveal region. Diagnosis of MacTel2 and associated macular neovascularization (MNV) was confirmed with fundus fluorescein angiography (FA), optical coherence tomography (OCT), and angiography (OCTA). The patient was treated with three bilateral injections of faricimab on a monthly basis. Following the last injection, there was improvement of best corrected visual acuity (BCVA), reduction of macular thickening on OCT, and shrinkage of the MNV on OCTA. This is the first reported case of usage of intravitreal injection of faricimab for the management of visual loss due to macular neovascularization due to MacTel2.

## Introduction

Macular telangiectasia is an acquired retinal vascular disease associated with progressive degeneration of the Müller cells [1,2]. It affects middle-aged patients with no gender predilection. It may be an asymmetric disease, which is characterized by loss of retinal translucency, intraretinal crystalline deposits, intraretinal migration of the retinal pigment epithelium (RPE), and abnormalities of perifoveal macular vessels. These vascular changes include telangiectasia of macular vessels, right-angled venules, vascular invasion of the foveal avascular zone, and macular neovascularization (intraretinal and subretinal) [2]. Optical coherence tomography (OCT) reveals macular thinning, the presence of hyporeflective intraretinal cavitations, disruption of the ellipsoid zone, outer retinal atrophy, the development of a full-thickness macular hole, and retinal neovascularization in the intraretinal and subretinal space [3,4].

Although initially thought to be a retinal vascular disease, MacTel2 is now believed to be due to degeneration of the Müller cells, which maintain the blood retinal barrier and provide trophic support to the surrounding neural tissue. This degeneration is associated with nutritional deprivation to the neurons and capillary endothelial cell dysfunction leading to atrophy and disorganization of the outer retina [5].

Macular neovascularization is often associated with acute visual loss and the occurrence of metamorphopsia. Unlike most of the causes of macular neovascularization, the neovascular process initiates in the intraretinal space instead of the choroidal vasculature. Marsonia K et al. showed that the incidence of MNV is 10.6% in eyes with MacTel2, and the mean development of MNV was at 2.36 years from baseline. Seventy-two percent of eyes with MNV had best-corrected visual acuity (BCVA) <20/40 [6]. Early treatment of MNV with anti-vascular endothelial growth factor (anti-VEGF) injections is associated with improved anatomical and visual outcomes [7].

Faricimab-svoa (Vabysmo™, Genentech, San Francisco, CA) is a bispecific antibody designed to neutralize both vascular endothelial growth factor-A (VEGF-A) and angiopoietin-2 (Ang-2) and synergistically promotes stabilization of retinal vasculature in retinal vascular disease [8]. It has been approved for the management of neovascular age-related macular degeneration, diabetic macular edema, and retinal vascular occlusion [8,9].

We report a case of MacTel2 associated with MNV, which was treated with intravitreal injection of faricimab. Following three injections of faricimab on a monthly basis, there was a reduction in the size of the neovascular complex and an improvement in BCVA.

## Case presentation

A 52-year-old lady of Asian descent presented with reduction of central vision for four weeks. She also complained of distorted vision, which had worsened since last week. There was no medical history of diabetes mellitus or any systemic illness. She reported that none of her family members had any similar ocular problems. She denied any history of chronic drug intake, and there was no relevant past medical and ocular history.

**Figure 1:**
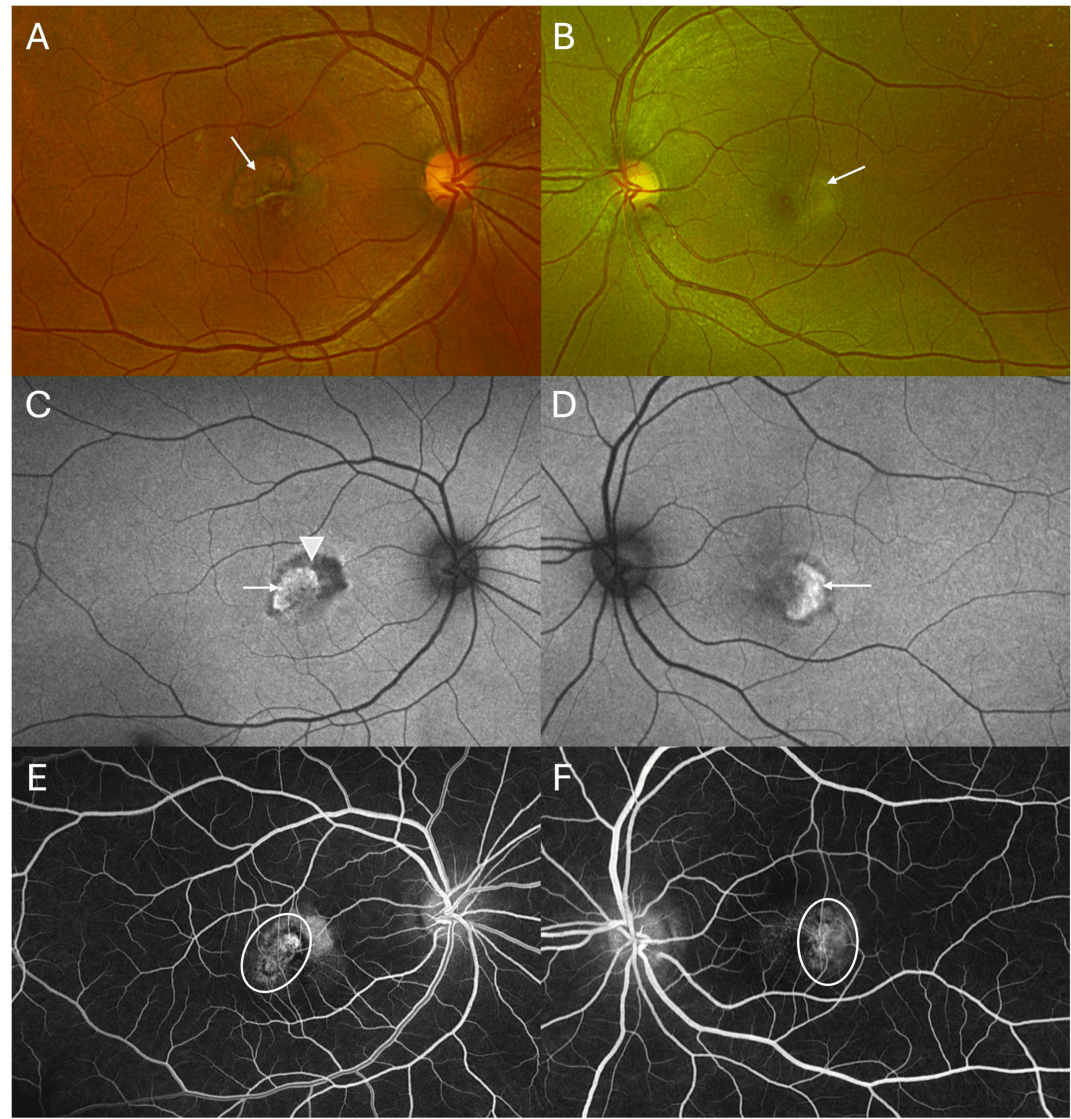
Fundus image, fundus autofluorescence image, and fluorescein angiography image of a patient with macular telangiectasia type 2 A color fundus photograph of the right eye (A) showed the presence of perifoveal telangiectasia, right-angled venules, and the presence of a circumscribed lesion with subretinal fibrosis (arrow) suggestive of macular neovascularization. The left eye (B) had subtle grayish discoloration around the macula (arrow), with telangiectasia and right-angled vessels and a more grayish lesion temporal to the fovea, suggestive of macular neovascularization. Fundus autofluorescence of the right eye (C) showed the presence of a hyperautofluorescent lesion (MNV) (arrow) surrounded by a hypoautofluorescent lesion indicative of RPE atrophy (arrowhead); the left eye (D) showed a bean-shaped area of hyperautofluorescent lesion suggestive of MNV (arrow). Fluorescein angiography of the right eye (E) demonstrated perifoveal telangiectatic vessels with transmission defect, confirming the presence of MNV (oval circle); the left eye (F) showed similar findings, confirming the presence of MNV (oval circle) in MacTel2.

On examination, her BCVA was 20/100 and 20/60 in the right and left eyes, respectively. Color vision with Ishihara pseudo-isochromatic plates was normal in both eyes. Pupillary examination and visual fields with confrontation tests were normal. The anterior segment was unremarkable. The intraocular pressures were 15 mmHg with Goldmann applanation tonometry. Dilated fundus examination of the right eye showed perifoveal telangiectasia, right-angled venules, and the presence of a circumscribed lesion with subretinal fibrosis suggestive of macular neovascularization (Figure 1A). The left eye had subtle grayish discoloration around the macula, with telangiectasia and right-angled vessels and a more grayish lesion temporal to the fovea suggestive of macular neovascularization (Figure 1B). Fundus autofluorescence of the right eye showed the presence of a hyperautofluorescent lesion (MNV) with a surrounded hypoautofluorescent lesion indicative of RPE atrophy (Figure 1C); the left eye showed a bean-shaped area of hyperautofluorescent lesion suggestive of MNV (Figure 1D).

Fundus fluorescein angiography of the right eye showed leakage in the late phases indicative of MNV, with perifoveal telangiectasia, right-angled vessels, and transmission defects (Figure 1E). The left eye had retinal telangiectasia and right-angled vessels. In the late stages, there was leakage noted in the temporal quadrant of the macula (Figure 1F).

**Figure 2:**
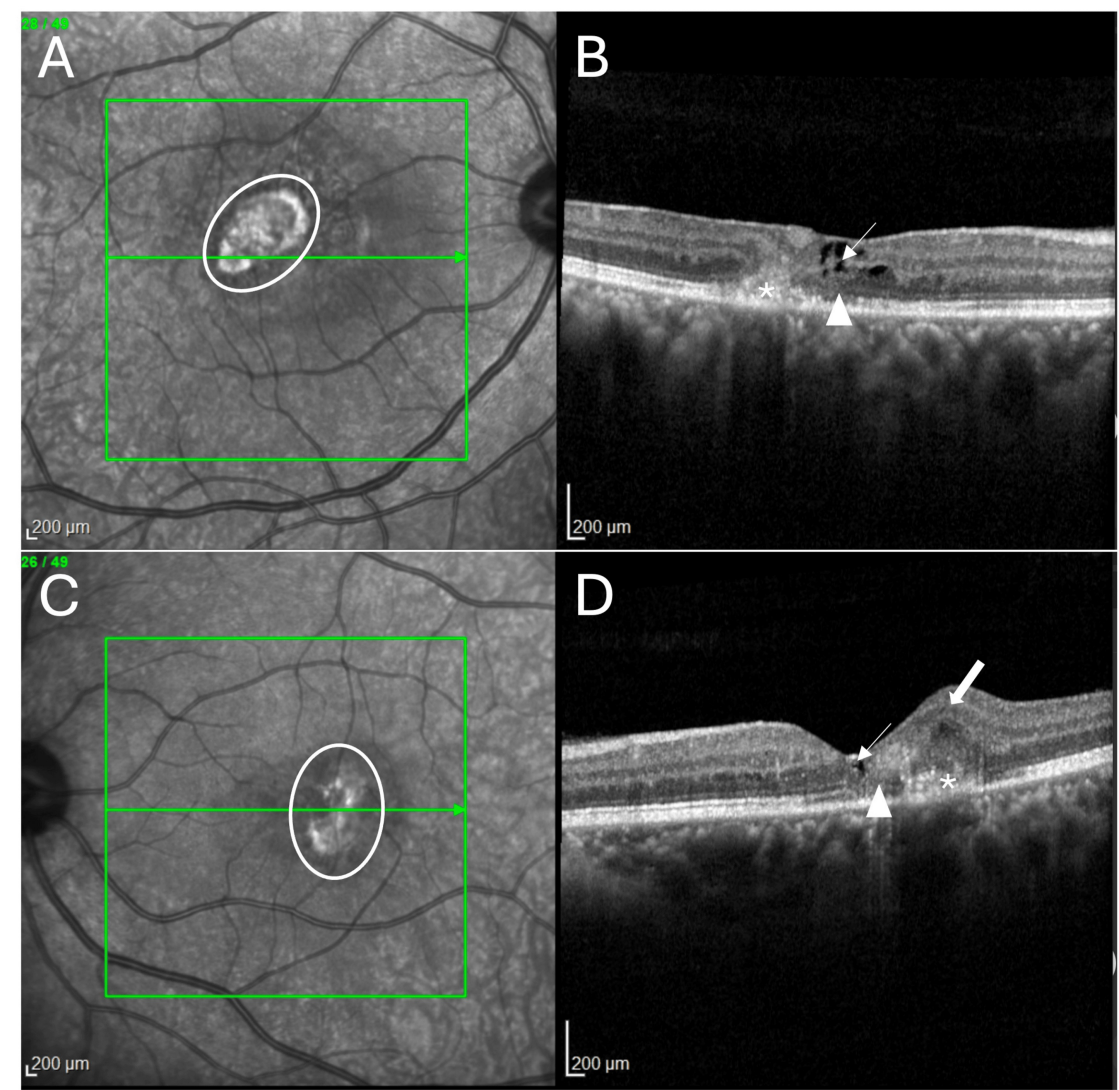
Near infrared reflectance (NIR) image and optical coherence tomography (OCT) image in macular telangiectasia type 2 (MacTel2) Near-infrared reflectance images of the right eye (A) and the left eye (C) revealed the presence of a hyperautofluorescent lesion indicative of MNV (oval circle). OCT of the right eye (B) and left eye (D) showed intraretinal cavitation (arrow), loss of external limiting membrane (ELM) and ellipsoid zone (arrowhead), and subretinal hyperreflective material temporal (asterisk) to the fovea (suggestive of MNV). The right eye (B) also showed the presence of the “ILM drape” sign (bold arrow), and the left eye (D) had increased macular thickening in the temporal quadrant (bold arrow). These features were diagnostic of MacTel2.

**Figure 3:**
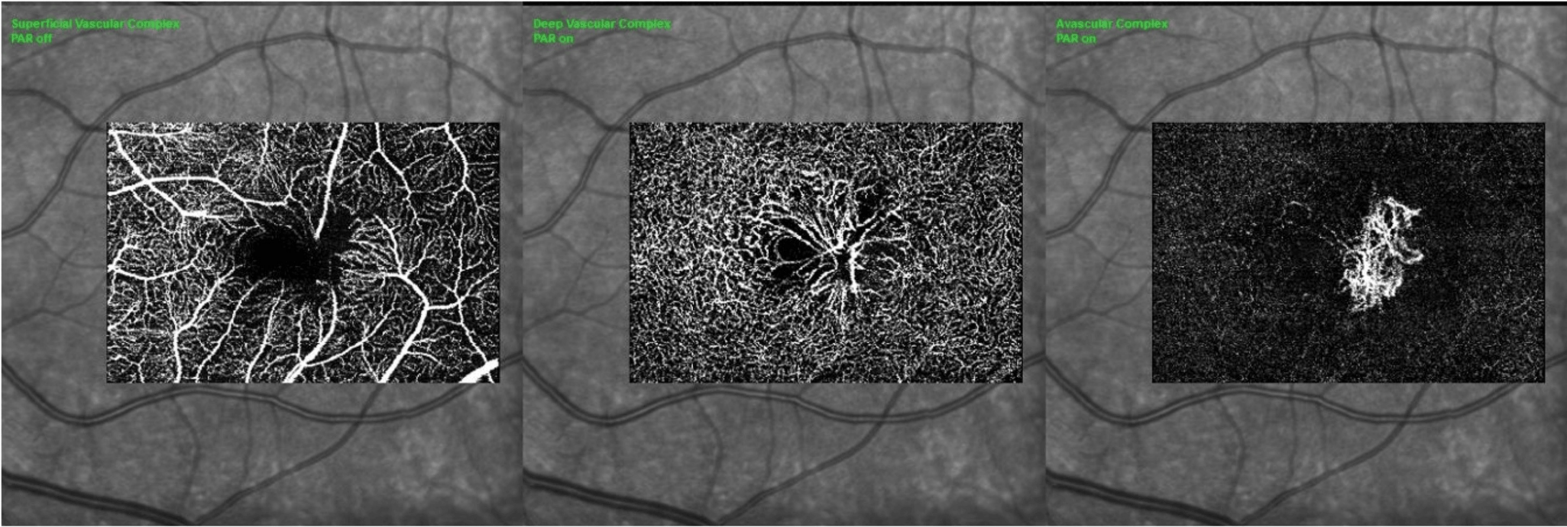
Optical coherence tomography angiography (OCTA) in macular telangiectasia type 2 OCT angiography in the right eye (A) and in the left eye (D) showed the presence of an irregular foveal avascular zone and a dipped right-angled venule (arrow) temporal to the foveal avascular zone in the superficial vascular complex. In the deep vascular complex, the right eye (B) and left eye (E) showed vascular invasion of the foveal avascular zone (arrow) and telangiectasia (arrowhead) in the perifoveal area. The avascular complex of the right eye (C) and of the left eye (F) demonstrated the presence of a neovascular network with multiple branches and abnormal anastomosis, which was evidence of active macular neovascularization (arrow).

OCT examination of the right eye displayed the presence of the ILM-drape sign, hyporeflective cavitation, loss of the ellipsoid zone, the external limiting membrane, and the presence of subretinal hyperreflective material (SHRM) with diffuse retinal thickness (Figure 2B). Similarly, the left eye showed hyporeflective cavitation, loss of the ellipsoid zone and external limiting membrane, and the presence of SHRM (Figure 2D). OCT angiography of both eyes demonstrated the presence of macular neovascularization in the avascular zone of the retina (pre-established map of the retinal vasculature between the outer plexiform layer and RPE) (Figures 3C, 3F). There were telangiectatic vessels in the deep capillary plexus with vascular invasion of the foveal avascular zone (Figures 3B, 3E). The neovascular net showed a ‘lacy wheel’ pattern with numerous small branching capillaries and anastomosis indicative of activity. Based on these findings, a diagnosis of macular neovascularization associated with MacTel2 was made.

The patient was recommended to undergo intravitreal injections of anti-VEGF medications. After discussing the pros and cons of the treatment, it was decided to treat her with intravitreal injection of faricimab. She underwent bilateral injections of faricimab (6 mg/0.05 mL of 120 mg/mL solution) on a monthly basis. Following every injection, the patient underwent a complete ophthalmic examination, including fundus photography, OCT examination, and OCT angiography. After each injection, there was progressive improvement of BCVA, along with reduction of macular thickness and size of the MNV on OCTA.

**Figure 4:**
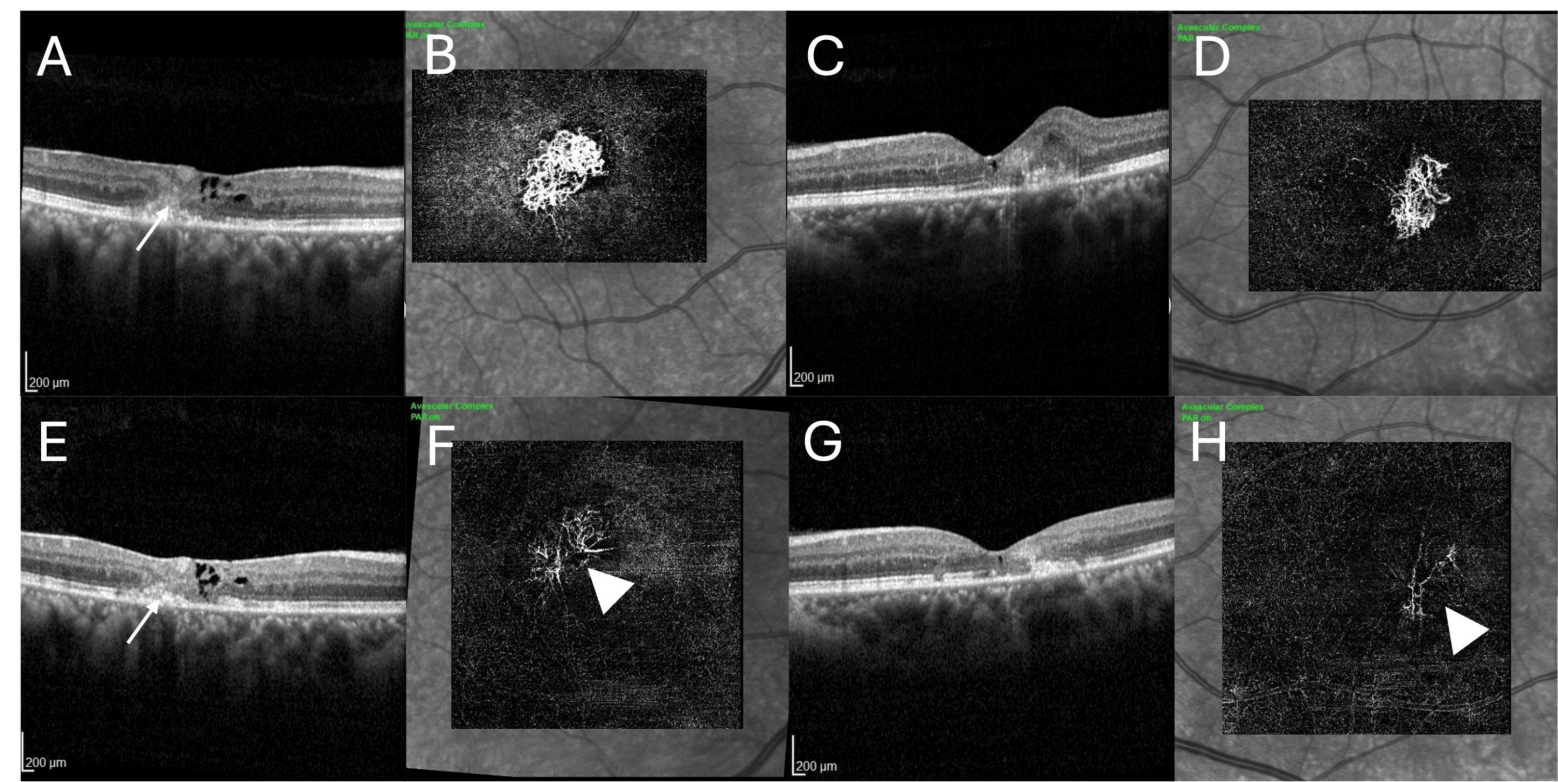
OCT and OCTA images in the post-injection period at three months and at eight months OCT: optical coherence tomography, OCTA: optical coherence tomography angiography The image demonstrates the changes in OCT and OCT angiography findings following three injections of faricimab (A, B, C, D) and at eight months of follow-up since presentation (E, F, G, H). In both eyes, there is a reduction of SHRM on OCT (arrow), right eye (A) and left eye (C) following the course of faricimab injections. OCTA revealed reduction of the neovascular complex in the right eye (B) and in the left eye (D). At eight months follow-up, both eyes continued to show signs of inactivity on OCT—right eye (E) and left eye (G). OCTA of the right eye (F) and of the left eye (H) displayed significant shrinkage of the MNV with a “dead tree” appearance (arrowhead) indicating quiescence.

During the treatment, there were no episodes of intraocular inflammation, no retinal vasculitis, and no retinal vascular occlusion. At the eight-month follow-up, the BCVA had improved to 20/25 in both eyes with no metamorphopsia. OCT showed reduction of macular thickness (Figure 4F) with shrinkage of MNV on OCTA with a “dead tree” appearance (Figure 4H). We found that intravitreal injection of faricimab was both effective and safe in the management of visual loss due to macular neovascularization associated with MacTel2.

## Discussion

MacTel2 is now considered a neurodegenerative disease in which patients experience progressive retinal atrophy that can significantly impact their functional vision and quality of life, especially reading speed and low-luminance vision, despite having only minimal changes in their visual acuity. Müller cells help maintain healthy neurons by secreting neurotrophic factors like ciliary neurotrophic factor (CNTF). As the Müller cells degenerate and deplete, the lack of trophic support to the photoreceptors and other retinal neurons leads to atrophy and disorganization of the outer retinal layers. The various vascular changes, such as telangiectasia and vascular dipping, are secondary to the neurodegeneration [5]. Also, degeneration of Müller cells leads to retinal thinning. With the progression of the disease, hypoxia occurs due to associated capillary endothelial cell dysfunction [2,10]. Finally, the combination of VEGF secretion and Müller cell dysfunction leads to intraretinal edema and subsequent development of macular neovascularization. The origin of MNV is presumed to be from the retinal vasculature, and the presence of intraretinal anastomosis has been demonstrated using indocyanine green angiography [10-12].

Yannuzzi et al. proposed a simplified classification for idiopathic juxtafoveal telangiectasia with two distinct types (Type I, or aneurysmal telangiectasia, and Type II, or perifoveal telangiectasia, also known as MacTel2). Mactel2 is mainly divided into two stages. The first stage is the non-proliferative stage, which is typified by gradual, progressive loss of vision due to foveal atrophy. The second stage is the proliferative stage, which is characterized by the development of MNV [2,10,11,13]. Several treatment options for the non-proliferative stage have been evaluated but have failed due to the progressive degenerative nature of this disease [13]. Recently, revakinagene taroretcel (revakinagene taroretcel-Iwey, Encelto™) received its first approval for the treatment of adults with idiopathic macular telangiectasia (MacTel2) in the USA. This single-dose intravitreal implant is an encapsulated cell-based gene therapy containing 200,000-400,000 allogeneic retinal pigment epithelial (RPE) cells expressing recombinant human ciliary neurotrophic factor (rhCNTF) [14].

Engelbrecht et al. revealed that the natural course of proliferative MacTel2 resulted in a very poor prognosis, including VA of 1/20 or less in 80% of the cases [15]. Although anti-vascular endothelial growth factor (anti-VEGF) agents may promote regression of abnormal neovascularization in eyes with proliferative MacTel2, therapeutic responses appear to vary significantly among different patient cohorts. Currently, there is a limited body of evidence directly comparing the safety and efficacy profiles of various anti-VEGF agents in the context of MacTel. While some studies have demonstrated favorable anatomical and functional outcomes with anti-VEGF treatment, others have failed to show significant improvements in visual acuity, underscoring the heterogeneous nature of treatment response in this condition [16-18]. Sriranganathan et al. published their systematic review, which evaluated the comparative efficacy and clinical outcomes of various intravitreal anti-vascular endothelial growth factor (anti-VEGF) therapies in patients with MacTEL2. The review included only 10 studies (eyes treated with bevacizumab, ranibizumab, and aflibercept) encompassing a total of 377 eyes. Overall, the review demonstrated favorable anatomical and functional outcomes in eyes treated with anti-VEGF agents compared to those managed with observation. The findings suggest a notable functional benefit of anti-VEGF therapy in the proliferative form of MacTel2, whereas evidence supporting its efficacy in non-proliferative MacTel2 remains inconclusive. Across studies, treatment with different anti-VEGF agents generally yielded comparable outcomes with a low incidence of adverse events [19].

Faricimab-svoa (Vabysmo™, Genentech, San Francisco, CA) is Food and Drug Administration (FDA) approved for the management of neovascular age-related macular degeneration, diabetic macular edema, and retinal vein occlusion [8,9]. Its novel bispecific dual-inhibition of VEGF-A and Ang-2 is thought to be associated with longer duration of action and better drying effect as compared to other anti-VEGFs in clinical trials. The patient, with active macular neovascularization due to proliferative MacTel2, was treated with intravitreal injection of faricimab. The injections were performed on a monthly basis, with each injection procedure followed by a complete ophthalmic examination, including fundus photography, OCT evaluation, and OCT angiography. The patient improved symptomatically with improvement of vision from 20/100 in the right eye and 20/60 in the left eye to 20/25 in both eyes. She was treated with three injections on a monthly basis. OCT of both eyes showed reduced macular thickening and reduction of subretinal hyperreflective material, while OCT angiography demonstrated a significant decrease in the size of the MNV. The patient did not experience any episode of intraocular inflammation, retinal vasculitis, or retinal vascular occlusion. This case report demonstrates the efficacy and safety of intravitreal faricimab in treating eyes with proliferative MacTel2. 

Anti-VEGF agents such as faricimab may be associated with favorable anatomical and functional outcomes, particularly in proliferative MacTel2; however, future large-scale clinical trials are warranted to assess the safety and efficacy of this novel molecule in this sight-threatening complication of an intriguing disease such as MacTel2. 

## Conclusions

Our case report demonstrates the role of intravitreal faricimab, which neutralizes both VEGF-A and Ang-2, in the management of macular neovascularization in macular telangiectasia type 2. OCT angiography plays an important role in diagnosing the presence, defining the extent, and demonstrating the regression of the neovascular complex following intravitreal therapy.
